# Compressive Response and Energy Absorption of Additively Manufactured Elastomers with Varied Simple Cubic Architectures

**DOI:** 10.3390/polym18030420

**Published:** 2026-02-05

**Authors:** Lindsey B. Bezek, Sushan Nakarmi, Jeffery A. Leiding, Nitin P. Daphalapurkar, Santosh Adhikari, Kwan-Soo Lee

**Affiliations:** 1 Los Alamos National Laboratory, Chemistry Division, Los Alamos, NM 87545, USA; lbezek@lanl.gov (L.B.B.); san_adh@lanl.gov (S.A.); 2 Los Alamos National Laboratory, Theoretical Division, Los Alamos, NM 87545, USA; snakarmi@lanl.gov (S.N.); jal@lanl.gov (J.A.L.); nitin@lanl.gov (N.P.D.); 3 Los Alamos National Laboratory, Materials Physics and Applications Division, Los Alamos, NM 87545, USA

**Keywords:** additive manufacturing, cellular materials, simple cubic, elastomer, compression, energy absorption

## Abstract

Additive manufacturing, and particularly the vat photopolymerization process, enables the fabrication of complex geometries at high resolution and small length scales, making it well-suited for fabricating cellular structures (e.g., foams and lattices). Among these, elastomeric cellular structures are of growing interest due to their tunable compliance and energy dissipation. However, comprehensive data on the compressive behavior of these structures remains limited, especially for investigating the structure-property effects from changing the density and distribution of material within the cellular structure. This study explores how the mechanical response of polyurethane-based simple cubic structures changes when varying volume fraction, unit cell length, and unit cell patterning, which have not been systematically investigated previously in additively manufactured elastomers. Increasing volume fraction from 10% to 50% yielded significant changes in compressive stress–strain performance (decreasing strain at 0.5 MPa by 41.6% and increasing energy absorption density by 3962.5%). Although changing the unit cell length between 2.5 and 7 mm in ~30 mm parts did not result in statistically different stress–strain responses, modifying the configuration of struts of different thicknesses across designs with 30% volume fraction altered the stress–strain behavior (differences of 12.5% in strain at 0.5 MPa and 109.4% for energy absorption density). Power law relationships were developed to understand the interactions between volume fraction, unit cell length, and elastic modulus, and experimental data showed strong fits (R^2^ > 0.91). These findings enhance the understanding of how multiple structural design aspects influence the performance of elastomeric cellular materials, providing a foundation for informing strategic design of tailorable materials for diverse mechanical applications.

## 1. Introduction

Additive manufacturing (AM) has enabled the fabrication of complex geometries such as cellular structures, which could be stochastic or periodic and include open-cell or closed-cell architectures [1]. Cellular structures produced by AM have the advantages of being lighter weight and lower density than the equivalent bulk structure, making them advantageous for applications requiring tunable capacities for strength-to-weight ratio, surface-to-volume ratio, stiffness, heat exchange, energy absorption, thermal insulation, and acoustic insulation. These traits make cellular structures valuable for the fabrication of lightweight and buoyant structures, structures undergoing vibration and shock, heat exchangers and sinks, sound absorbers, filters, packaging, electrodes, support structures, and biomedical implants [2,3].

A significant portion of the body of work for additively manufactured cellular structures is associated with rigid materials [3,4,5,6,7], but there have been some promising advances related to the development and characterization of various additively manufactured elastomeric cellular structures [8,9]. Elastomeric cellular structures have the potential to improve helmet performance while maintaining comfort and breathability [10]. Graded elastomers could be integrated into soft robotic systems, biomedical supports for rehabilitation, or wearable electronic devices. Aerospace, defense, and packaging industries could benefit from enhanced lightweight solutions offering reusable impact resistance and more customizable performance compared to what is achievable with conventional packing foams. Continued study into additively manufactured elastomeric cellular structures will advance understanding of their multifunctional behavior to accelerate their implementation in practical applications.

Several researchers have used lattices to demonstrate the feasibility of printing complex geometries with a new elastomeric material, such as styrene-butadiene rubber latex [11], extremely soft elastomers [12], and silicones modified with either reinforced thiol-ene [13], silica filler [14,15], or polytetrafluoroethylene [16]. The incorporation of a liquid crystal elastomer that experiences a mechanically-induced phase transition with large strains has been used to increase energy absorption [17], and a deep learning model has been used to generate elastic auxetic metamaterials [18].

One of the AM techniques commonly employed for manufacturing elastomeric cellular structures is vat photopolymerization, during which liquid material in a vat is selectively cured with radiant light exposure. One common method, commonly termed stereolithography or SLA, involves the use of a rastering laser to initiate photocuring of a part in a layer-by-layer fashion.

Using this AM technique, in previous work, the authors studied Formlabs Elastic 50A, a commercial polyurethane-based elastomer containing two acrylate monomers and a photoinitiator [19], to better understand the different modes of buckling across nine different unit cell (UC) types, for which the UC designs were based on either struts or triply periodic minimal surfaces [20]. Simulations of the quasi-static compression tests supported the experimental results, where it could be seen that the mechanical response and energy density can be tailored for different damping applications based on UC type [20]. In a subsequent study, the simple cubic (SC), body-centered, and gyroid topologies, which were identified to exhibit varied deformation behaviors [20], were further investigated through simulations and physical experiments to analyze the effects of part size, displacement rate, and aging on the compression response. The main findings included that increasing part size while maintaining the same volume fraction (VF) and UC length shifted the densification region for the body-centered structure, the highest tested compression rate led to slightly stiffer stress–strain responses, and there were no substantial changes in compressive response across five weeks [21].

Although these studies provide insights into key considerations for designing accurate, consistent, and durable elastomeric cellular structures, they were limited to 20% VF. While the effects of VF on the mechanical properties of rigid cellular structures have been heavily studied [22,23,24], the effects of VF on the mechanical properties of elastomeric cellular structures have not been largely studied. Only a couple of studies have investigated printing structures with thermoplastic polyurethane or similar materials with different VFs, where it could be seen that increasing VF increased elastic modulus [25,26], stiffness [27], and in another study, compressive stress at 10% and 50% strain, compressive modulus, and energy absorption, all following power law fits [28].

In addition to VF, another aspect to consider when designing cellular elastomers is how the material is allocated for a specific VF and part size. Modifying the distribution of mass across a cellular elastomer can redistribute an applied load, leading to tunable compressive behavior. It has been previously shown that varied UC designs that have the same VF and part size have the potential to yield unique compressive responses for elastomers [20]. When considering the same UC type, designers could apply uniform changes (e.g., adapting UC length and strut thickness) or non-uniform approaches (e.g., functional grading/manipulation of strut thickness or UC length) [29,30,31,32]. These design strategies have been shown to modulate mechanical response to different degrees for rigid cases. For Ni-Ti, decreasing UC length in 20% VF gyroids decreased manufacturing fidelity but did not largely affect the stress–strain response, though the smallest UC length resulted in a decreased maximum strain [33]. Decreasing UC length in Ti-6Al-4V body-centered cubic tensile bars of 36% VF led to higher ultimate tensile strength and elastic modulus [34]. Decreasing UC length in CuAlMn gyroids (15% VF) and AlMgScZr gyroids (20% VF) decreased manufacturing accuracy but increased compressive strength and modulus [35,36]. Decreasing UC length in 316 L stainless steel body-centered cubic structures caused higher stiffness and energy absorption [37]. For polylactic acid, decreasing UC length in 20% VF gyroids caused higher elastic stiffness [38]. Offsetting rows of SC UCs altered the global structure buckling behavior and reduced the compressive strength and energy absorption in nylon [39]. There has also been research incorporating multiple UC lengths within a single part, where nylon Schwarz-P and gyroid structures were graded between 3 × 3 and 9 × 9 UC patterns, though there were no major changes in the compressive response nor energy absorption due to the graded design [40]. In another study, when UC length was modified between 2 mm and 20 mm in nylon SC structures, there was an increase in compressive strength with smaller UC lengths. A plateau maximum compressive strength was observed for UC lengths of 2 mm, 2.2 mm, and 2.5 mm for part sizes of 20 mm × 20 mm × 20 mm, and this strength could also be reached with different combinations of UC length and part size (e.g., 10 mm UC length for a 60 mm × 60 mm × 60 mm part size) [41].

While these studies demonstrate the importance of understanding the interactions between UC length and patterning for designing parts with tunable compressive performance, it may not be possible to directly correlate these results to the behavior of elastomers because the deformation behavior could be different. For example, elastomeric SC structures have been shown to experience global structural buckling [20]. Although global buckling can occur in rigid parts [42], it is less common since rigid SC structures typically have failure initiation points at a corner [41]. The lack of studies on how these elements of part design affect compression response and energy absorption significantly limits the knowledge base for structure-property relationships for elastomeric cellular structures. In this study, the authors explore how the interplay of design decisions with an SC architecture affects the compressive stress–strain behavior and energy absorption capacity for a commercial elastomeric material. With their heat resistance, toughness, and durability, polyurethanes are useful in several applications [20]; thus, Elastic 50A is selected for study. This also enables part performance from new part designs to be directly compared to previous studies to assess how design can modify and enhance compression behavior. The interaction between VF, UC length, and elastic modulus is explored through power law relationships that are derived from existing fits that were previously applied to rigid cellular structures. In the present study, an experimental investigation of the SC structure is conducted to complement the previous studies with simulation and experiment [20,21]. While theory and simulation provide critical insight into the mechanisms of localized buckling and lattice response, they rely on assumptions that may not effectively account for potential effects from geometric imperfections, boundary compliance, or other unforeseen interactions. Experiments offer a complementary role in providing direct measurements of the system response under physically realistic conditions, thus having the potential to inform and guide the refinement of simulations. The novelty of this work is the comparison in performance of different combinations of VFs, UC lengths, and UC patterns for an elastomeric material. Advancing knowledge of the compressive response for elastomeric cellular structures will enable more predictable performance by guiding material design for tunable mechanical response.

## 2. Materials and Methods

**Figure 1:**
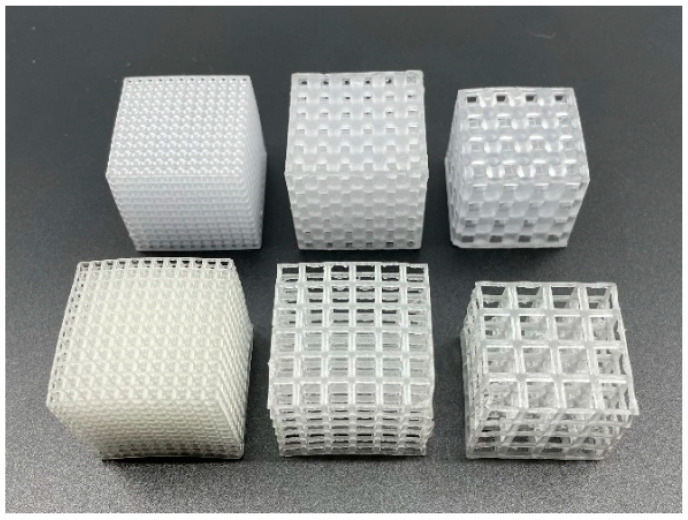
Sample cellular structures investigated in this study include (**top row**) 50% VF with 2.5, 5, and 7 mm UCs and (**bottom row**) 10% VF with 2.5, 5, and 7 mm UCs. The first print layer of the parts (i.e., in the XY plane) corresponds to the bottom surface, and the build (Z) direction is up.

**Figure 2:**
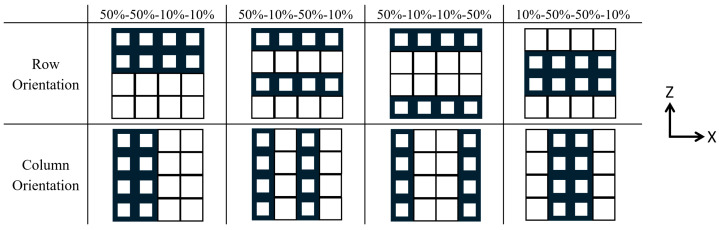
Schematic illustrating the SC structures with 30% VF that are designed by patterning rows with 10% and 50% VF UCs. The structures were tested in both row orientation and column orientation. The Z direction corresponds to the build direction, and each individual part layer is in the XY plane.

The parametric design platform in Grasshopper (Rhinoceros 7 plugin) was used to generate the SC structures. Design parameters were then selected to ensure that struts were thick enough to be successfully fabricated and that pores were large enough for effective cleaning. To investigate the effect of VF on compressive properties, it was necessary to control UC length and patterning. Parts were designed with 10% and 50% VFs using 2.5 mm UCs patterned 12 × 12 × 12, 5 mm UCs patterned 6 × 6 × 6, and 7 mm UCs patterned 4 × 4 × 4 (Figure 1). These designs also enable the investigation of the effect of UC length on compressive properties for the two VFs. Additionally, cellular structures with 35% VF using 2.5 mm UCs patterned 12 × 12 × 12 were designed. Finally, to probe further into the effects of UC design, 4 × 4 × 4 cellular structures with 7 mm UCs were designed with 30% VF by modifying different rows to have 10% or 50% VFs (Figure 2). A summary of all designed parts is provided in Table 1.

Parts were printed on a Formlabs Form 3+ (Somerville, MA, USA) using Formlabs Elastic 50A material (Somerville, MA, USA). The Form 3+ uses 405 nm optical wavelength and an 85 µm laser spot size [43]. Parts had 100 µm layers and no supports. Early layer exposure for the first four layers was reduced to 350 mJ/cm^2^ to reduce overprinting, and all other exposure settings were the software defaults (55 mJ/cm^2^ for perimeter and 49 mJ/cm^2^ for interior). Following previously used post-processing procedures [21], when parts were finished printing, they were removed from the build plate and submerged in isopropyl alcohol (IPA) for 10 min with gentle agitation to promote removal of the residual uncured material. The parts were then rinsed in deionized (DI) water, submerged again in IPA for 10 min, and rinsed again in DI water. The parts were then submerged in DI water and placed in an ultraviolet (UV) curing oven (Formlabs Form Cure, Somerville, MA, USA) for five min to remove any surface tackiness. Finally, parts were dried with compressed air before being placed in an oven for 20 min at 60 °C.

A Qualitest densimeter MD-300S (Plantation, FL, USA) was used to measure dry mass and submerged mass of each sample to determine the volume of water displaced. VF could then be calculated using the outer dimensions of each sample to represent a theoretical solid volume.

An Instron 3343 (Norwood, MA, USA) with a 1 kN load cell was used for compression testing. Parts were oriented such that the first print layer was flush with the bottom compression platen. The 30% VF structures were also tested with a 90° rotation such that the alternating rows were oriented as vertical columns. The selected testing procedure was guided by ASTM D575-91 and consistent with previous studies’ procedures [20,21]. The cellular structures were compressed at 12 mm/min to 0.5 MPa for three cycles, and data were recorded on the last cycle. The 10%—2.5 and 35%—2.5 samples were additionally tested at 120 mm/min and 1000 mm/min to evaluate any rate dependence in the material performance. To maintain consistency in analyzing the nominal stress–strain data, the zero strain point was selected when the parts were pre-stressed to 0.001 N. Stresses were calculated by dividing the applied load by the samples’ cross-sectional area, for which the outer dimensions were used. Samples were tested in triplicate. Compressive strains at 0.5 MPa were calculated, and one-way analysis of variance (ANOVA) was performed using a 95% confidence interval to test for statistically significant differences in means across the samples using the Real Statistics Resource Pack for Microsoft Excel. For all tests, the null hypothesis of all means being equal was rejected, and a Tukey’s Honestly Significant Difference post-hoc test was then performed to conduct pairwise comparisons, for which two means are statistically different when a q statistic is greater than a computed critical q value.

When a cellular structure is compressed, its energy absorption can be expressed per unit volume. This energy absorption density for the cellular structures, W(ε), is computed as the area under the stress–strain curve:

(1) $$W\left(\varepsilon \right)=\int_{0}^{\varepsilon }\sigma \left(\varepsilon \right)d\varepsilon ,$$

where σ is stress and ε is strain. Typically, the selected strain value is at the onset of densification, $\varepsilon_{d}$, which is at the end of the plateau region of the stress–strain curve. This can be estimated at the point at which the energy absorption efficiency is at a maximum. It has previously been shown that the efficiency parameter, η(ε), can be effectively estimated as the energy density up to a certain strain divided by the peak stress up to that strain, $\sigma_{p}\left(\varepsilon \right)$ [20,44]:

(2) $$\eta \left(\varepsilon \right)=\frac{\int_{0}^{\varepsilon }\sigma \left(\varepsilon \right)d\varepsilon }{\sigma_{p}\left(\varepsilon \right)}.$$

The previous equations emphasize strain-resolved energy absorption metrics. To complement this perspective, analysis of global energy absorption based on the total energy absorbed over the loading stroke is also considered [45]. The effective stroke, $S_{ef}$, is defined as the displacement, u, which corresponds to $\varepsilon_{d}$:

(3) $$S_{ef}=\varepsilon_{d}\times h,$$

where h is part height. The total energy absorption, EA, can then be computed as

(4) $$EA=\int_{0}^{S_{ef}}F\;du,$$

where F is the compression load. The specific energy absorption, SEA, is defined as

(5) $$SEA=\frac{EA}{m},$$

where m is the sample mass. The global energy absorption efficiency, $\eta_{global}$, can then be calculated as

(6) $$\eta_{global}=\frac{EA}{h\times F_{u=S_{ef}}},$$

## 3. Results and Discussion

In this section, an analysis of the printed cellular structures is first presented, including a discussion on dimensional integrity and manufacturing constraints. Stress–strain results from the compression tests are then analyzed, followed by the evaluation of energy absorption density and efficiency. Finally, the authors assess the effectiveness of applying power law fits from the literature to relate VF, geometric UC parameters, and part properties to better understand the interaction effects and aid future efforts for designing cellular structures with tailored mechanical response.

### 3.1. Analysis of Printed Parts

**Figure 3:**
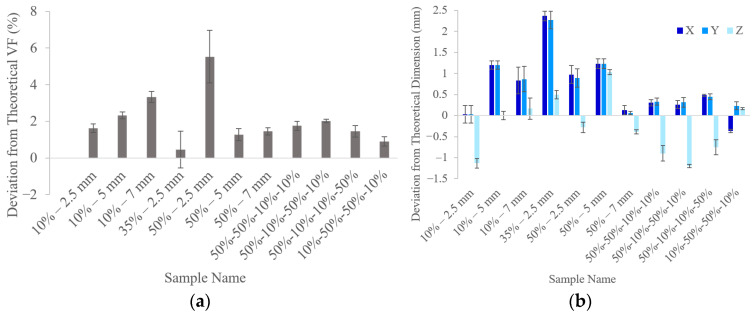
Deviations in (**a**) VF and (**b**) outer dimensions between the printed parts and the theoretical values. There were three samples of each type. Error bars represent one sample standard deviation.

The printed parts’ VFs were measured and compared to the designed values. As shown in Figure 3a, on average, all parts were slightly overbuilt in terms of VF. The 50%—2.5 samples were, on average, 5.5% higher in VF than designed, which was due to excess material stuck in a few of the innermost pores. In future studies, to compensate for this overprinting, the printer’s radiant exposure or exposure time could be lowered, or digital parts could be scaled smaller in compensation. The lowered print quality for the 50%—2.5 samples is understandable, as similar behavior has been observed with metal lattices, where increased specific surface area has led to worse forming quality because of excess metal powder adhesion [35,36].

Figure 3b shows the deviations of the outer dimensions of the samples. In about half of the samples, the Z dimension (height) was smaller than the target, but typically not more than ~1 mm. Unlike the XY dimensions, which are controlled by the radiant exposure, the Z dimension is controlled by mechanical constraints between the build plate and vat, and elastomers could be prone to relaxation after printing once those constraints are removed. Although the 50%—2.5 mm samples showed the highest deviation in VF, these parts did not have substantially different deviations in outer dimensions, supporting the conclusion that the trapped internal material is the cause of the higher VF. On the other hand, the 35%—2.5 mm samples had the highest deviation in outer dimensions, with the lengths and widths being ~2 mm larger than expected. Interestingly, these parts were the closest to the target VF. This further demonstrates how VF is not solely dependent on the parts’ outer dimensions but also on the strut geometry. For example, in the 10% VF cases, thin struts are highly sensitive to overprinting, leading to thicker-than-designed struts despite closer to nominal external dimensions, resulting in higher than intended VFs. Conversely, in the 35%—2.5 mm samples, dimensional errors are primarily from a global scaling of the lattice geometry rather than a local increase in strut thickness.

Although the selected AM system has an 85 µm laser spot size [43], it is possible that, compared to rigid materials, compliant materials achieve lower XY resolution due to the material’s freedom to flex and relieve stress in the printed layers while Z direction forces are applied during further layer fabrication. It is suspected that this led to thinner struts periodically being misaligned or fully detached, though these were inconsistent occurrences that were resolved in reprints. This compliance also likely contributes to the slight curvatures often witnessed in the global shape of the cellular structures. Measurements were taken by a single person to minimize operator-dependent variability when handling the elastomers, and if the dimensions were not consistent across the length of a part side, the measurement reflected a representative estimate.

Another consideration for lower geometric accuracy is that the SC architecture has unsupported overhangs. Supports would be ideal to reinforce the overhangs, but it would not be possible to access and remove internal supports. Soluble supports are an effective alternative; however, the selected vat photopolymerization system only accommodates one material. Visual inspection of the structures did not reveal significant defects in the overhanging struts.

### 3.2. Mechanical Testing

**Figure 4:**
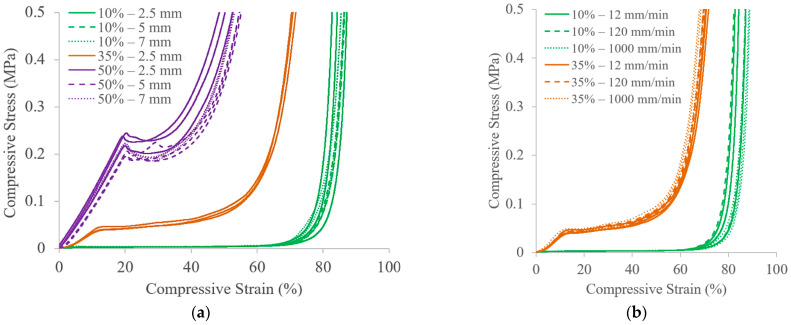

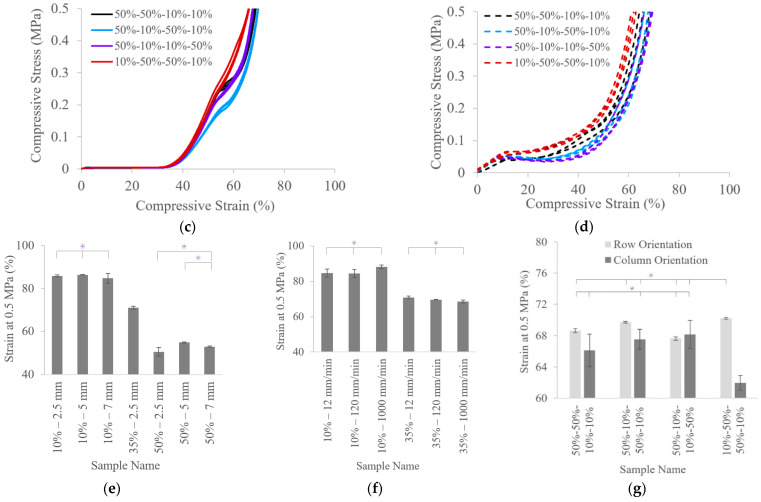
Compressive stress–strain plots for investigating different (**a**) VFs and UC lengths, (**b**) compression testing rates using cellular structures with 2.5 mm UCs, (**c**) row orientations, and (**d**) column orientations. The strains at the maximum stress are summarized in plots for the cellular structures of different (**e**) VFs and UC lengths, (**f**) compression testing rates, and (**g**) row and column orientations. Error bars represent one sample standard deviation. Asterisks (*) indicate the pairwise comparisons that are not statistically significant at a 95% confidence interval (e.g., for 10% VF, the values for the 2.5 mm, 5 mm, and 7 mm cases are not significantly different when comparing against each other, but they are statistically different from all other cases).

Figure 4 shows the stress–strain results from all compression tests, and supplemental tables for the statistical analysis can be found in the Appendix A. The comparison of performance for different VFs and UC lengths can be found in Figure 4a, with the corresponding maximum strains provided in Figure 4e. The stress–strain curves show that the selection of VF largely dictates the deformation behavior. The 10% VF samples do not experience an initial rise in stress, and densification begins at ~60% strain. This can be attributed to the low density of the structure, which collapses more readily when the load is applied. The 35%—2.5 mm samples show clear definition between the initial linear elastic region (from 0% to ~10% strain), the plateau region (from ~10% to ~50% strain), and the densification region (above ~50% strain). In contrast, the 50% VF samples exhibit higher stiffness through a longer and steeper linear region (from 0% to ~20% strain), and densification begins at ~40% strain. As the VF increases, there are statistically significant decreases in the strain at 0.5 MPa due to the denser structures being able to absorb more energy. However, varying the UC length between 2.5 mm and 7 mm does not have significant effects on the compressive performance of the 10% and 50% VF structures. The average strains for the 50%—2.5 mm samples are slightly lower than those with larger UCs at the same VF, and this could be due to the effects of their slightly higher VF (Figure 3a). In comparison to previous work, the stress–strain curve for SC structures using the same material with 20%—5 mm samples falls between those of the 10% VF and 35% VF samples, where the 20%—5 mm samples experienced a linear region plateauing at ~0.025 MPa and densification spanning from ~52% to ~80% [21].

Figure 4b presents the stress–strain results for the rate study with the corresponding maximum strains provided in Figure 4f. The curvatures of the stress–strain profiles at the higher rates are nearly identical to those of the 12 mm/min testing condition, and the maximum strains measured for each of the rates are not statistically different from each other for each VF. These results also make sense within the context of previous work, where the strains at 0.5 MPa were ~76–78% for the 20%—5 mm samples across the same tested rates [21]. Although the materials were expected to have rate-dependent performance, the tested compression rates all fall within the quasi-static domain. Testing at strain rates higher than the quasi-static region, which has been performed in a recent study for this material, has shown evidence of a shift to a wave-dominated deformation mechanism for the SC architecture [46].

Although modifying the UC length for the same VF was not shown to highly influence the compressive response, another study was completed to evaluate if this were also true for scenarios with non-uniform mass distribution. Thus, parts were designed with tailored placement of UCs of different thicknesses. To achieve parts with 30% VF, equal amounts of UCs with 10% or 50% VF were placed in different designated rows of 4 × 4 × 4 structures (Figure 2). The results for the structures tested in row orientations are shown in Figure 4c, and the results for the structures tested in column orientations are shown in Figure 4d. The corresponding maximum strains are provided in Figure 4g. The stress–strain trends for the structures tested in row orientations indicate a blend of the behavior for the 10% and 50% VF cases. The linear elastic region is delayed during the collapse of the 10% VF rows, and there is no initial rise in stress until ~30%, after which the stress–strain trend mimics that of the 50% VF samples in Figure 4a with a linear region and a short plateau prior to densification.

**Figure 5:**
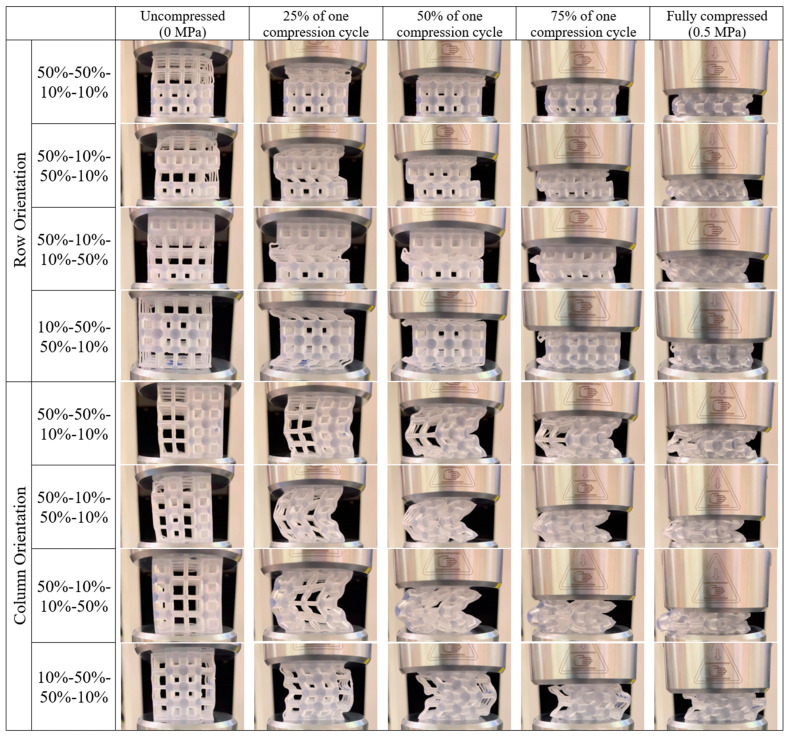
Images of the 30% VF structures during the progression of a compression cycle. Since the strains at 0.5 MPa vary by structure, images are relative to the compression cycle.

Despite all row orientation cases sharing the same approximate point of initial stress rise and similar final strains (~68–70%), the intermediate stress–strain behavior is design dependent. Most notably, the 50%-10%-50%-10% case experiences relatively lower stress compared to the other cases when strained ~40–60%. The reason for this can be seen in the images of the compressed samples (Figure 5), where the evolution of the compression behavior is shown for each sample across five images. At the maximum compression point, which is dictated by the samples undergoing a stress of 0.5 MPa, the samples exhibit slightly different strains, so the progression of images is portrayed by the percentage of each sample’s compression cycle between 0 and 0.5 MPa. About halfway into the compression cycle, all of the row orientation cases show a nearly full collapse of the 10% VF UCs, while the 50% VF UCs remain uncompressed. However, for the 50%-10%-50%-10% case, the buckling of the 10% VF rows results in the 50% VF rows being offset, such that the uniaxial load is not applied uniformly across the 50% VF UCs. Consequently, the load is applied across a smaller cross-sectional area, resulting in relatively lower forces until the UCs have collapsed, after which the bulk material volume dictates the densification behavior. This is an interesting finding that reinforces the opportunity for tailorable compressive response based on intentional design decisions.

Another noteworthy observation in Figure 4c is the 10%-50%-50%-10% case, which has consistently higher stresses than the other samples after ~50% strain. This may be attributed to the 50% VF UC rows being oriented both together and in the center of the sample. A higher stiffness can be seen in Figure 5 at 75% of the compression cycle, where the 10%-50%-50%-10% case is the only row orientation sample with 50% VF UCs mostly undeformed under the same load. The adjoining rows of 50% VF UCs provide stability, but what distinguishes the 10%-50%-50%-10% sample from the 50%-50%-10%-10% sample is that the latter has the collapsed 10% VF UC rows at only one platen boundary. For the 10%-50%-50%-10% case, the collapsed 10% VF UC rows are against both platens, which may have added compliance to both boundary constraints and introduced instability. In fact, after being stressed to 0.5 MPa, the 10%-50%-50%-10% case resembles more of local cell buckling behavior, while the other three samples show clear global structural buckling. It has previously been demonstrated that non-uniform or compliant constraints can promote localized buckling modes rather than long-wavelength buckling instability predicted by classical theory [47]. Similarly, Timoshenko’s theory of beams on elastic foundations demonstrates that an elastic foundation introduces a characteristic length scale that can suppress global buckling in favor of short-wavelength buckling modes [48]. At the earliest stage of compression of the 50% VF struts (~75% of the compression cycle), the 10%-50%-50%-10% sample behaves almost rigidly: the struts are nearly straight, and deformation is negligible because the initial axial stiffness of the struts dominates. The somewhat compliant platens only allow minor displacements, so the lattice effectively resists the applied load. As compression increases, localized instabilities begin to emerge in individual struts due to the combined effects of the part geometry and the platen-lattice interaction acting as an effective elastic foundation. This triggers localized, short-wavelength cell-scale buckling, and because of how the struts are constrained, it is possible that axial deformation contributes alongside bending, producing a stiffer stress–strain response than those of the other loading orientations that exhibit global Euler-type buckling.

Compared to the results from the row orientations, the stress–strain results for the column orientations (Figure 4d) all exhibit an initial linear region, a longer plateau region, and a wider spread in final strains (~62–68%). The shape of the stress–strain curve is typical for global structural buckling cases [20], which can also be observed in Figure 5 for all four structures. In column orientation, all UCs in the structures are immediately under load, with the 50% VF UCs controlling the direction of the buckling. There is less stability in the parts as they are compressed, especially for the 50%-10%-50%-10% and 50%-10%-10%-50% cases, where it can be observed that halfway through the compression cycle, the faces flush with the compression platens show tendencies to curl. On the other hand, the structures with the 50% VF columns side-by-side (50%-50%-10%-10% and 10%-50%-50%-10%) are more reinforced. These structures show less deformation from buckling at 25% through a compression cycle, which can be attributed to a stiffer response, as these structures tended to have the lower strains at 0.5 MPa. However, due to high sample variability in the 50%-50%-10%-10% samples, only the final strain for the 10%-50%-50%-10% case in column orientation is statistically lower than those of all other cases.

### 3.3. Energy Absorption Density

**Figure 6:**
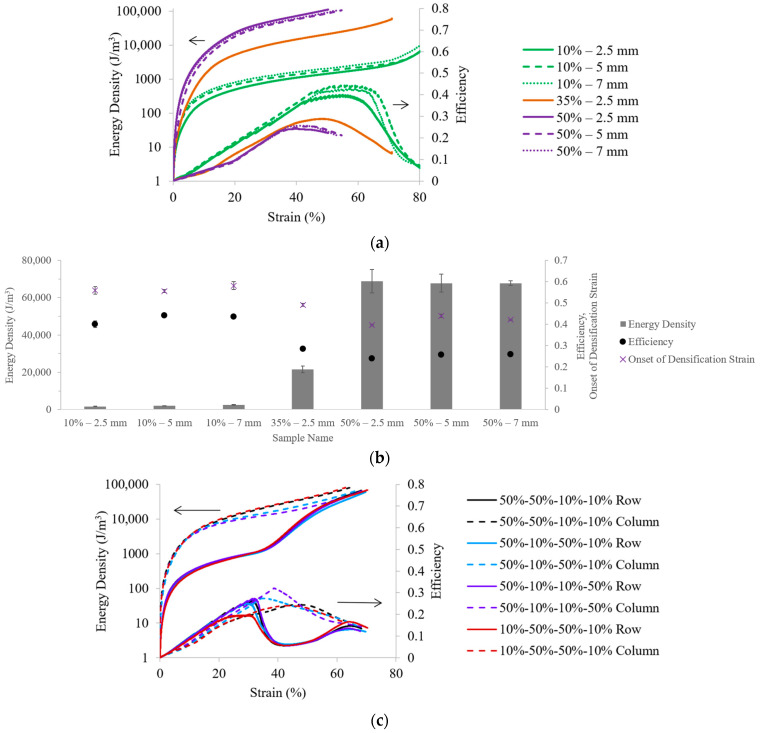

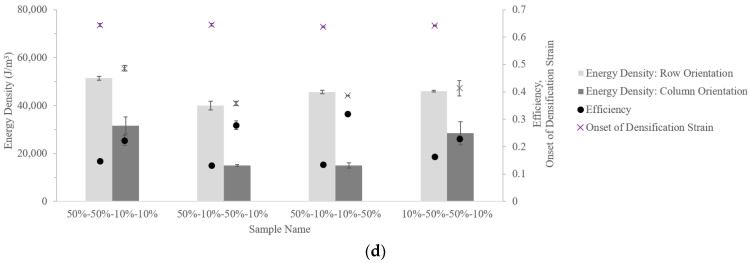
Comparisons of energy density and efficiency as functions of strain for representative cellular structures of (**a**) different VFs and UC lengths, and (**c**) different orientations. Calculated energy density based on the area of the stress–strain curve up to the onset of densification for the cellular structures of (**b**) different VFs and UC lengths, and (**d**) different orientations. Error bars represent one sample standard deviation.

The strain-resolved energy absorption density for the 10%, 35%, and 50% VF structures of different UC lengths is shown as a function of strain in Figure 6a, and the efficiency parameters are provided in the same plot. Figure 6b outlines the energy densities for each structure calculated up to the onset of densification strain, which is the strain at the point of maximum efficiency. As expected, parts with higher VFs are capable of absorbing more energy, with 50% VF samples having energy densities of ~70 kJ/m^3^, compared to 35% VF samples (~20 kJ/m^3^) and 10% VF samples (~2 kJ/m^3^). It can also be noted that the UC length does not affect the structures’ energy density.

The peak efficiencies for the 10% VF samples (~40%) are higher than those of the 50% VF samples (~25%) because the energy densities relative to the peak stresses are higher for the lower VF samples. However, because the 10% VF samples do not experience an initial linear region, their energy densities are considerably lower compared to those of the other VFs, which further supports how the struts in the 10% VF samples provided little resistance and immediately collapsed under load. The onset of densification strain also decreases with increasing VF, which aligns with the literature for other polymers [38].

Figure 6c shows the energy densities and efficiency parameters as functions of strain for the 30% VF structures. The row orientation structures have bimodal efficiency plots, indicative of the 10% VF UCs reacting to the load, followed by the 50% VF UCs. The first peak (~30% strain) marks the transition where the 10% VF UCs have collapsed, and appreciable stress starts to be measured in a delayed linear region. Thus, the performance of the structures is better represented by using the second efficiency peak (~65% strain), which is the transition between the short plateau region and densification.

The energy densities calculated up to the onset of densification strain for the 30% VF structures are provided in Figure 6d. For the row orientations, the 50%-50%-10%-10% samples have the highest energy density, while onset of densification strains and efficiency parameters are comparable across all samples. The 50%-10%-50%-10% samples, as expected, have the lowest energy density due to the reduced absorption capacity from the offset rows. For the column orientations, the 50%-50%-10%-10% and 10%-50%-50%-10% cases have higher energy density, which aligns with the findings of these structures exhibiting more stiff behavior from the adjacent 50% VF UC columns. Comparing orientations, the row orientation tests result in higher energy absorption, higher onset of densification strains, and lower efficiency parameters. It can also be seen that comparing the results between Figure 6b,d, many of the 30% VF structures, including all of the row orientations, have higher energy densities than that of the 35%—2.5 mm samples, indicating how non-uniform material distribution can enable a structure to exhibit improved mechanical performance compared to the homogenous equivalent. As a result, it would be interesting in future work to explore this design space further, supported by numerical analysis to validate experiments with simulations and advance knowledge of design strategies to achieve the desired compressive response.

**Figure 7:**
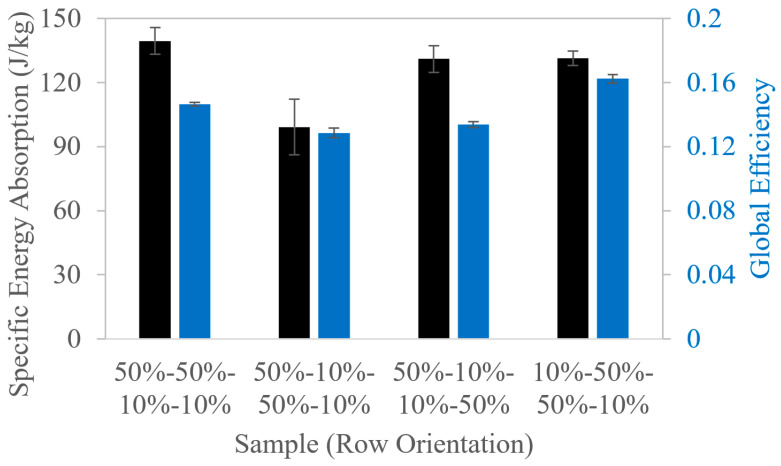
Global specific energy absorption and energy absorption efficiency for the SC structures with 30% VF that were designed by patterning rows with 10% and 50% VF UCs. Error bars represent one sample standard deviation.

The strain-resolved energy absorption metrics identify key transitions during the evolution of deformation, but they do not directly quantify overall performance or mass efficiency. Thus, global metrics are additionally reported in Figure 7 for the row orientation samples to provide complementary perspectives on cellular structure performance and further demonstrate how several design parameters, including VF and UC configuration, have the potential to affect the global part deformation mechanisms. The global specific energy absorption reflects the mass efficiency of the cellular structure to absorb energy. The 50%-50%-10%-10% case has the highest specific energy absorption, while the 50%-10%-50%-10% case has the lowest specific energy absorption, which is consistent with the trends for strain-resolved energy density (Figure 6d). The global energy absorption efficiency reflects how effectively the load is supported by the cellular structure during deformation. For these samples, global energy absorption efficiency is lower than strain-resolved efficiency because the former compares the total absorbed energy to a theoretical maximum for which the cellular structure sustains the load at the effective stroke uniformly across the sample height. In elastomeric cellular structures, stress is not evenly distributed, so not all elements carry peak loads throughout the compression cycle. In contrast, strain-resolved efficiency evaluates energy absorption relative to the local stress at each strain increment, which better highlights the heterogeneous load-sharing characteristic among struts in the elastomeric cellular structures.

**Figure 8:**
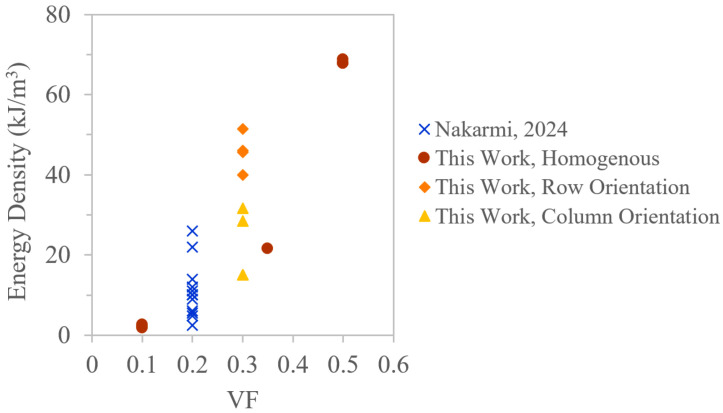
Summary of experimentally validated energy absorption densities across different VFs, where specific part design is demonstrated to enable tunable performance [20].

Figure 8 provides a summary plot of energy absorption density versus VF for the parts in this study as well as previous work using the same material system. In prior work, it was shown that for the same VF, energy density can be modulated through UC selection, which changed deformation mechanisms and energy absorption efficiency [20]. This study built on that work, isolating designs to the SC architecture, where it can be seen that with homogenous part designs, VF has a strong effect on energy density. Further, the 30% VF parts demonstrate that a wide range of energy densities can be achieved simply by configuring the placement of UCs.

### 3.4. Interaction of VF, UC, and Part Properties

After studying the effects of lattice design parameters on the elastic modulus of rigid body-centered cubic tensile bars, Maskery and co-authors proposed a power law relationship to relate UC parameters with the relative elastic modulus, E*, which is defined as the cellular structure’s modulus as a fraction of the modulus of the equivalent solid part [34]. Starting with the semi-empirical scaling relationship proposed by Gibson and Ashby [49]:

(7) $$E*=A\times VF^{B},$$

where VF is volume fraction or relative density and A and B are constants, they recognized that additional UC parameters also follow a power law trend, which led them to propose a modified form:

(8) $$E*=A\times VF^{B}\times {v*}^{\;C},$$

where C is a constant and v* is the relative cell volume, which is defined as the volume of a single UC (i.e., UC length cubed) as a fraction of the exterior dimensions encompassing the patterned UCs. (For example, v* for the 10%—2.5 mm case is (2.5 mm)^3^ ÷ (30 mm)^3^). They chose to work with v* instead of absolute measurements of UC length [35] or cell volume, so that general rules could be developed for how a cellular structure of a particular material performs compared to the solid equivalent [34]. This parameter enables the capturing of the effects of changing UC length and patterning for a set part size and VF, which was evaluated in their study as well as the current study. The application of Equation (8) was not within the scope of the work in which it was proposed, and here, it is of interest to evaluate its appropriateness in being applied to the current work’s data, for which other properties could be substituted into the model forms of Equations (7) and (8).

The effects of VF, v*, and their interaction on several properties (E*, energy absorption density ($W(\varepsilon_{d})$), and strain at 0.5 MPa) were evaluated using a factorial regression in log-log space. E* was calculated using ~4.29 MPa as the elastic modulus of the solid equivalent, which was derived from previous work where solid cubes of the same material and size were tested in compression with similar testing parameters [21].

For each of the properties, the interaction terms were not significant (*p* > 0.05); therefore, they were removed, and each regression was rerun following the form of Equation (8). For each of the properties, C was then found to not be a statistically significant coefficient, so a final regression was run without v*, and thus following the form of Equation (7). The constants for the regressions following both Equations (7) and (8) are provided in Table 2 along with the calculated coefficient of determination (R^2^) values (statistical tables are provided in Appendix A). Although v* was not found to be significant, it is still provided in the analysis to compare the shape of the response, even given its weak effect in this study. It can be seen that the magnitudes of the C coefficients are small, indicating a negligible contribution, but their inclusion shifts the values of A due to shared variance among the predictors. While the inclusion of v* results in a slightly higher R^2^ for the E* fit, since C is not statistically significant, this increase likely reflects overfitting of the data from a small sample size. The reduced model is preferred, and the consistency of the values of B indicates the properties’ strong dependence on VF.

The power law fits were previously applied to metal materials of different cellular structure types, and this study offers promising initial verification that they are applicable across broader material classes and cellular designs. For the E* fit, the value of B falls in the range of previously reported values, given that Gibson and Ashby report B to be ~2 for bending-dominated structures, while multiple researchers report it closer to 1 for stretching-dominated structures, such as the SC architecture [49,50]. Additionally, Gibson and Ashby report A to be between 0.1 and 4.0 based on metal foam data [49,50], and the computed A values also fall into this range for the E* fit.

**Figure 9:**
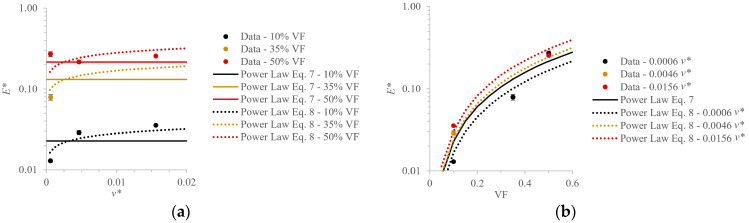

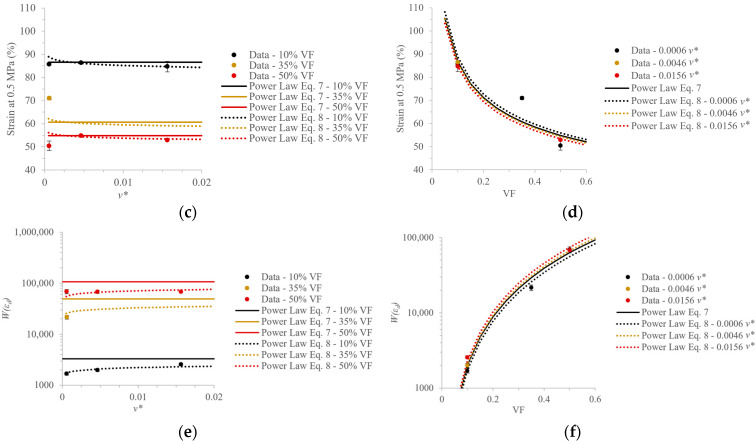
Power law fits of the form of Equations (7) and (8) applied to the experimental results of (**a**,**b**) relative elastic modulus, (**c**,**d**) strain at 0.5 MPa, and (**e**,**f**) strain-resolved energy absorption density. Plots are provided for comparing the properties with relative cell volume (in (**a**,**c**,**e**)) and VF (in (**b**,**d**,**f**)).

E* is plotted as a function of v* in Figure 9a and as a function of VF in Figure 9b. When VF is fixed, E* shows no consistent trend with increases in v*, further supporting its statistical insignificance for the selected material and part designs. However, when v* is fixed, as VF increases, E* increases. Strain at 0.5 MPa (Figure 4e) is plotted as a function of v* in Figure 9c and as a function of VF in Figure 9d. For this property, the two power law fits nearly overlap due to C being near zero. Figure 9e and f show strain-resolved energy absorption density as functions of v* and VF, respectively, where a strong alignment of the power law fits to the data is observed. Although the high R^2^ values in Table 2 indicate strong regressions, the plots in Figure 9 indicate that there is still improvement needed to appropriately capture the experimental variability. It is recognized that the experimental data collected in this study are from a small sample size, and more data would be desirable to verify these initial findings and ensure that the expressions are applicable over wide ranges of VF and v*.

## 4. Conclusions

Elastomeric SC structures were designed with varying VFs, UC lengths, and UC configurations and fabricated with vat photopolymerization AM to study how design parameters affect parts’ compressive properties and energy absorption density. The novel contributions of this work include (1) the experimental investigation of structure-property relationships for elastomeric cellular architectures with UCs of varying lengths, strut thicknesses, and configurations; and (2) the investigation of the interplay of multiple design parameters that influence the elastomer’s mechanical properties. This work provides reference data for which future parts requiring performance within this material property window can be guided by the selected configurations, with confidence that material response will be predictable and consistent with the observed results. The main findings are summarized here:

- When VF increases while maintaining the same UC length and approximate part size, the strut thickness increases. For the tested SC elastomeric structure, VF largely dictates the magnitude and duration of the elastic, plateau, and densification regions in the compressive stress–strain response. High VFs led to lower strains at 0.5 MPa (41.6% decrease between 10% and 50% VF) due to the added mass stiffening the parts. This is especially seen in the energy density, which increases by 3962.5% between the 10% and 50% VF samples.
- When UC length increases while maintaining the same VF and approximate part size, the number of patterned UCs decreases. Increasing UC size in the tested elastomers did not result in significant changes in the stress–strain response nor the energy density. A power law regression with interaction terms was performed, and VF was the only variable to have a statistically significant effect on the part properties.
- Non-homogenous samples with 30% VF were obtained by combining equal amounts of UCs with 10% and 50% VF. When these UCs of varied strut thickness were assembled into diverse configurations of rows and columns, it resulted in unique stress–strain responses attributed to specific part designs, as revealed by the visual observation of the unique collapsing behaviors during the compression cycle. Percentage differences in strain at 0.5 MPa were up to 12.5%, and percentage differences in energy density were up to 109.4%. This suggests that even though VF highly influences the compressive behavior, the distribution of material, especially in non-homogeneous ways, also factors into the mechanical performance. This is a promising result for cellular elastomers to potentially achieve a broader spectrum of mechanical responses by considering an extended design space of more organic strategies, such as functionally graded lattices and stochastic foams.

With the flexibility to carefully tune the design of foams and lattices, AM enables the fabrication of customizable lightweight elastomers with controllable compressive response and energy absorption for a wide range of applications, including vibrational damping, thermal management, and filtration. While this study was limited to the SC architecture to focus on UC designs, it would be of interest to study other elastomeric cellular structures and materials in the future, supplemented by simulations of the compressive response to validate the experimental data.

## Figures and Tables

**Table 1:** Summary of cellular structures investigated in this study.

Sample Name	Volume Fraction (%)	Unit Cell Length (mm)	Unit Cells Patterned	Side Length (mm)
10%—2.5 mm	10	2.5	12 × 12 × 12	30
10%—5 mm	10	5	6 × 6 × 6	30
10%—7 mm	10	7	4 × 4 × 4	28
35%—2.5 mm	35	2.5	12 × 12 × 12	30
50%—2.5 mm	50	2.5	10 × 10 × 10	30
50%—5 mm	50	5	6 × 6 × 6	30
50%—7 mm	50	7	4 × 4 × 4	28
50%-50%-10%-10%	30	7	4 × 4 × 4	28
50%-10%-50%-10%	30	7	4 × 4 × 4	28
50%-10%-10%-50%	30	7	4 × 4 × 4	28
10%-50%-50%-10%	30	7	4 × 4 × 4	28

**Table 2:** Constants and R^2^ values for the power law equations that are fit to the experimental data. Only the models in the form of Equation (7) have statistically-supported coefficients.

	Model Form: Equation (7)			Model Form: Equation (8)		
	$E^{*}=A\times VF^{B}$	$\varepsilon_{\sigma =0.5\;\mathrm{MPa}}=A\times VF^{B}$	$W(\varepsilon_{d})=A\times VF^{B}$	$E^{*}=A\times VF^{B}\times {v*}^{\;C}$	$\varepsilon_{\sigma =0.5\;\mathrm{MPa}}=A\times VF^{B}\times {v*}^{\;C}$	$W(\varepsilon_{d})=A\times VF^{B}\times {v*}^{\;C}$
A	0.57	45.08	277,316.00	1.75	41.27	472,806.00
B	1.40	−0.28	2.14	1.43	−0.29	2.15
C	N/A	N/A	N/A	0.18	−0.01	0.09
R^2^	0.90	0.91	0.99	0.95	0.91	0.99

## Data Availability

Data is available upon request.

## Appendix A. Statistical Tables

Table A1. Comparing means of strain at 0.5 MPa for the homogenous samples compressed at 12 mm/min
Table A2. Comparing means of strain at 0.5 MPa for the 30% VF row/column orientation samples
Table A3. Comparing means of strain at 0.5 MPa for the homogenous samples compressed at higher rates
Table A4. Power law coefficients for relative elastic modulus
  (i) All effects tested
  (ii) Interaction removed (Model form Equation (8))
  (iii) *v** removed (Model form Equation (7))
Table A5. Power law coefficients for strain at 0.5 MPa
  (i) All effects tested
  (ii) Interaction removed (Model form Equation (8))
  (iii) *v** removed (Model form Equation (7))
Table A6. Power law coefficients for strain-resolved energy density
  (i) All effects tested
  (ii) Interaction removed (Model form Equation (8))
  (iii) *v** removed (Model form Equation (7))

**Table A1:** Comparing means of strain at 0.5 MPa for the homogenous samples compressed at 12 mm/min.

ANOVA: Single Factor									
DESCRIPTION					Alpha	0.05			
Group	Count	Sum	Mean	Variance	SS	Std Err		Lower	Upper
10%—2.5 mm	3	257.2875	85.7625	0.279544	0.559087	0.692681		84.27685	87.24815
10%—5 mm	3	259.1817	86.3939	0.017417	0.034835	0.692681		84.90825	87.87955
10%—7 mm	3	254.2341	84.7447	5.170337	10.34067	0.692681		83.25905	86.23035
35%—2.5 mm	3	212.9794	70.99313	0.410522	0.821043	0.692681		69.50748	72.47879
50%—2.5 mm	3	151.357	50.45233	4.05636	8.11272	0.692681		48.96668	51.93799
50%—5 mm	3	164.44	54.81333	0.093776	0.187552	0.692681		53.32768	56.29899
50%—7 mm	3	158.6797	52.89323	0.047996	0.095992	0.692681		51.40758	54.37889
ANOVA									
Sources	SS	df	MS	F	p value	Eta-sq		RMSSE	Omega Sq
Between Groups	4916.313	6	819.3855	569.2463	6.75 × 10^−16^	0.995918		13.77493	0.993878
Within Groups	20.1519	14	1.439422						
Total	4936.465	20	246.8233						
TUKEY HSD/KRAMER			alpha	0.05					
group	mean	n	ss	df	q-crit				
10%—2.5 mm	85.7625	3	0.559087						
10%—5 mm	86.3939	3	0.034835						
10%—7 mm	84.7447	3	10.34067						
35%—2.5 mm	70.99313333	3	0.821043						
50%—2.5 mm	50.45233333	3	8.11272						
50%—5 mm	54.81333333	3	0.187552						
50%—7 mm	52.89323333	3	0.095992						
		21	20.1519	14	4.829				
Q TEST									
group 1	group 2	mean	std err	q-stat	lower	upper	*p*-value	mean-crit	Cohen d
10%—2.5 mm	10%—5 mm	0.6314	0.692681	0.91153	−2.71356	3.976357	0.994	3.344957	0.526272
10%—2.5 mm	10%—7 mm	1.0178	0.692681	1.469363	−2.32716	4.362757	0.936	3.344957	0.848337
10%—2.5 mm	35%—2.5 mm	14.76937	0.692681	21.32203	11.42441	18.11432	0.000	3.344957	12.31028
10%—2.5 mm	50%—2.5 mm	35.31017	0.692681	50.97607	31.96521	38.65512	0.000	3.344957	29.43105
10%—2.5 mm	50%—5 mm	30.94917	0.692681	44.68025	27.60421	34.29412	0.000	3.344957	25.79615
10%—2.5 mm	50%—7 mm	32.86927	0.692681	47.45223	29.52431	36.21422	0.000	3.344957	27.39656
10%—5 mm	10%—7 mm	1.6492	0.692681	2.380893	−1.69576	4.994157	0.636	3.344957	1.374609
10%—5 mm	35%—2.5 mm	15.40077	0.692681	22.23356	12.05581	18.74572	0.000	3.344957	12.83655
10%—5 mm	50%—2.5 mm	35.94157	0.692681	51.8876	32.59661	39.28652	0.000	3.344957	29.95732
10%—5 mm	50%—5 mm	31.58057	0.692681	45.59178	28.23561	34.92552	0.000	3.344957	26.32243
10%—5 mm	50%—7 mm	33.50067	0.692681	48.36376	30.15571	36.84562	0.000	3.344957	27.92283
10%—7 mm	35%—2.5 mm	13.75157	0.692681	19.85266	10.40661	17.09652	0.000	3.344957	11.46194
10%—7 mm	50%—2.5 mm	34.29237	0.692681	49.50671	30.94741	37.63732	0.000	3.344957	28.58271
10%—7 mm	50%—5 mm	29.93137	0.692681	43.21088	26.58641	33.27632	0.000	3.344957	24.94782
10%—7 mm	50%—7 mm	31.85147	0.692681	45.98287	28.50651	35.19642	0.000	3.344957	26.54822
35%—2.5 mm	50%—2.5 mm	20.5408	0.692681	29.65405	17.19584	23.88576	0.000	3.344957	17.12077
35%—2.5 mm	50%—5 mm	16.1798	0.692681	23.35822	12.83484	19.52476	0.000	3.344957	13.48587
35%—2.5 mm	50%—7 mm	18.0999	0.692681	26.1302	14.75494	21.44486	0.000	3.344957	15.08628
50%—2.5 mm	50%—5 mm	4.361	0.692681	6.295826	1.016043	7.705957	0.008	3.344957	3.634897
50%—2.5 mm	50%—7 mm	2.4409	0.692681	3.523843	−0.90406	5.785857	0.233	3.344957	2.034492
50%—5 mm	50%—7 mm	1.9201	0.692681	2.771982	−1.42486	5.265057	0.477	3.344957	1.600405

**Table A2:** Comparing means of strain at 0.5 MPa for the 30% VF row/column orientation samples.

ANOVA: Single Factor												
DESCRIPTION							Alpha		0.05			
Group		Count	Sum		Mean	Variance	SS		Std Err		Lower	Upper
R50%-50%-10%-10%		3	205.8979		68.63263	0.064971	0.129941727		0.650388		67.25387	70.01139
R50%-10%-50%-10%		3	209.0857		69.69523	0.012507	0.025014907		0.650388		68.31647	71.07399
R50%-10%-10%-50%		3	202.8405		67.6135	0.046463	0.09292686		0.650388		66.23474	68.99226
R10%-50%-50%-10%		3	210.6138		70.2046	0.011215	0.0224295		0.650388		68.82584	71.58336
C50%-50%-10%-10%		3	198.3122		66.10407	4.328942	8.657884527		0.650388		64.72531	67.48283
C50%-10%-50%-10%		3	202.5582		67.5194	1.632587	3.26517318		0.650388		66.14064	68.89816
C50%-10%-10%-50%		3	204.4418		68.14727	3.222115	6.444229307		0.650388		66.76851	69.52603
C10%-50%-50%-10%		3	185.8649		61.95497	0.833296	1.666592287		0.650388		60.57621	63.33373
ANOVA												
Sources		SS	df		MS	F	*p* value		Eta-sq		RMSSE	Omega Sq
Between Groups		139.6288	7		19.94698	15.71851	4.44395 × 10^−6^		0.873046		2.288996	0.811068
Within Groups		20.30419	16		1.269012							
Total		159.933	23		6.95361							
TUKEY HSD/KRAMER					alpha	0.05						
group	mean			n	ss	df	q-crit					
R50%-50%-10%-10%	68.63263333			3	0.129942							
R50%-10%-50%-10%	69.69523333			3	0.025015							
R50%-10%-10%-50%	67.6135			3	0.092927							
R10%-50%-50%-10%	70.2046			3	0.02243							
C50%-50%-10%-10%	66.10406667			3	8.657885							
C50%-10%-50%-10%	67.5194			3	3.265173							
C50%-10%-10%-50%	68.14726667			3	6.444229							
C10%-50%-50%-10%	61.95496667			3	1.666592							
				24	20.30419	16	4.896					
Q TEST												
group 1	group 2			mean	std err	q-stat	lower	upper		*p*-value	mean-crit	Cohen d
R50%-50%-10%-10%	R50%-10%-50%-10%			1.0626	0.650388	1.633795	−2.1217	4.246898		0.934	3.184298	0.943272
R50%-50%-10%-10%	R50%-10%-10%-50%			1.019133	0.650388	1.566963	−2.16516	4.203431		0.946	3.184298	0.904687
R50%-50%-10%-10%	R10%-50%-50%-10%			1.571967	0.650388	2.416969	−1.61233	4.756264		0.682	3.184298	1.395438
R50%-50%-10%-10%	C50%-50%-10%-10%			2.528567	0.650388	3.887784	−0.65573	5.712864		0.177	3.184298	2.244613
R50%-50%-10%-10%	C50%-10%-50%-10%			1.113233	0.650388	1.711646	−2.07106	4.297531		0.917	3.184298	0.988219
R50%-50%-10%-10%	C50%-10%-10%-50%			0.485367	0.650388	0.746273	−2.69893	3.669664		0.999	3.184298	0.430861
R50%-50%-10%-10%	C10%-50%-50%-10%			6.677667	0.650388	10.26721	3.493369	9.861964		0.000	3.184298	5.927777
R50%-10%-50%-10%	R50%-10%-10%-50%			2.081733	0.650388	3.200758	−1.10256	5.266031		0.368	3.184298	1.847959
R50%-10%-50%-10%	R10%-50%-50%-10%			0.509367	0.650388	0.783174	−2.67493	3.693664		0.999	3.184298	0.452166
R50%-10%-50%-10%	C50%-50%-10%-10%			3.591167	0.650388	5.521579	0.406869	6.775464		0.022	3.184298	3.187885
R50%-10%-50%-10%	C50%-10%-50%-10%			2.175833	0.650388	3.345441	−1.00846	5.360131		0.319	3.184298	1.931491
R50%-10%-50%-10%	C50%-10%-10%-50%			1.547967	0.650388	2.380068	−1.63633	4.732264		0.697	3.184298	1.374133
R50%-10%-50%-10%	C10%-50%-50%-10%			7.740267	0.650388	11.90101	4.555969	10.92456		0.000	3.184298	6.871049
R50%-10%-10%-50%	R10%-50%-50%-10%			2.5911	0.650388	3.983932	−0.5932	5.775398		0.158	3.184298	2.300124
R50%-10%-10%-50%	C50%-50%-10%-10%			1.509433	0.650388	2.320821	−1.67486	4.693731		0.721	3.184298	1.339927
R50%-10%-10%-50%	C50%-10%-50%-10%			0.0941	0.650388	0.144683	−3.0902	3.278398		1.000	3.184298	0.083533
R50%-10%-10%-50%	C50%-10%-10%-50%			0.533767	0.650388	0.82069	−2.65053	3.718064		0.999	3.184298	0.473826
R50%-10%-10%-50%	C10%-50%-50%-10%			5.658533	0.650388	8.700248	2.474236	8.842831		0.000	3.184298	5.023091
R10%-50%-50%-10%	C50%-50%-10%-10%			4.100533	0.650388	6.304753	0.916236	7.284831		0.007	3.184298	3.640051
R10%-50%-50%-10%	C50%-10%-50%-10%			2.6852	0.650388	4.128615	−0.4991	5.869498		0.133	3.184298	2.383657
R10%-50%-50%-10%	C50%-10%-10%-50%			2.057333	0.650388	3.163242	−1.12696	5.241631		0.381	3.184298	1.826299
R10%-50%-50%-10%	C10%-50%-50%-10%			8.249633	0.650388	12.68418	5.065336	11.43393		0.000	3.184298	7.323215
C50%-50%-10%-10%	C50%-10%-50%-10%			1.415333	0.650388	2.176138	−1.76896	4.599631		0.777	3.184298	1.256394
C50%-50%-10%-10%	C50%-10%-10%-50%			2.0432	0.650388	3.141511	−1.1411	5.227498		0.389	3.184298	1.813752
C50%-50%-10%-10%	C10%-50%-50%-10%			4.1491	0.650388	6.379427	0.964802	7.333398		0.007	3.184298	3.683164
C50%-10%-50%-10%	C50%-10%-10%-50%			0.627867	0.650388	0.965373	−2.55643	3.812164		0.996	3.184298	0.557358
C50%-10%-50%-10%	C10%-50%-50%-10%			5.564433	0.650388	8.555565	2.380136	8.748731		0.000	3.184298	4.939558
C50%-10%-10%-50%	C10%-50%-50%-10%			6.1923	0.650388	9.520938	3.008002	9.376598		0.000	3.184298	5.496916

**Table A3:** Comparing means of strain at 0.5 MPa for the homogenous samples compressed at higher rates.

ANOVA: Single Factor												
DESCRIPTION							Alpha		0.05			
Group		Count	Sum		Mean	Variance	SS		Std Err		Lower	Upper
10%—12 mm/min		3	254.2341		84.7447	5.170337	10.34067		0.843997		82.90579	86.58361
10%—120 mm/min		3	253.6077		84.5359	5.590483	11.18097		0.843997		82.69699	86.37481
10%—1000 mm/min		3	264.6875		88.22917	0.947751	1.895502		0.843997		86.39026	90.06808
35%—12 mm/min		3	212.9794		70.99313	0.410522	0.821043		0.843997		69.15422	72.83204
35%—120 mm/min		3	209.1308		69.71027	0.015819	0.031638		0.843997		67.87136	71.54918
35%—1000 mm/min		3	205.9512		68.6504	0.687032	1.374064		0.843997		66.81149	70.48931
ANOVA												
Sources		SS	df		MS	F	p value		Eta-sq		RMSSE	Omega Sq
Between Groups		1193.581	5		238.7163	111.7068	1.24 × 10^−9^		0.978967		6.102097	0.968506
Within Groups		25.64389	12		2.13699							
Total		1219.225	17		71.71913							
TUKEY HSD/KRAMER					alpha	0.05						
group	mean			n	ss	df	q-crit					
10%—12 mm/min	84.7447			3	10.34067							
10%—120 mm/min	84.5359			3	11.18097							
10%—1000 mm/min	88.22916667			3	1.895502							
35%—12 mm/min	70.99313333			3	0.821043							
35%—120 mm/min	69.71026667			3	0.031638							
35%—1000 mm/min	68.6504			3	1.374064							
				18	25.64389	12	4.75					
Q TEST												
group 1	group 2			mean	std err	q-stat	lower	upper		*p*-value	mean-crit	Cohen d
10%—12 mm/min	10%—120 mm/min			0.2088	0.843997	0.247394	−3.80018	4.217784		1.000	4.008984	0.142833
10%—12 mm/min	10%—1000 mm/min			3.484467	0.843997	4.128532	−0.52452	7.49345		0.103	4.008984	2.383609
10%—12 mm/min	35%—12 mm/min			13.75157	0.843997	16.29339	9.742583	17.76055		0.000	4.008984	9.406994
10%—12 mm/min	35%—120 mm/min			15.03443	0.843997	17.81338	11.02545	19.04342		0.000	4.008984	10.28456
10%—12 mm/min	35%—1000 mm/min			16.0943	0.843997	19.06915	12.08532	20.10328		0.000	4.008984	11.00958
10%—120 mm/min	10%—1000 mm/min			3.693267	0.843997	4.375926	−0.31572	7.70225		0.078	4.008984	2.526442
10%—120 mm/min	35%—12 mm/min			13.54277	0.843997	16.046	9.533783	17.55175		0.000	4.008984	9.264161
10%—120 mm/min	35%—120 mm/min			14.82563	0.843997	17.56599	10.81665	18.83462		0.000	4.008984	10.14173
10%—120 mm/min	35%—1000 mm/min			15.8855	0.843997	18.82176	11.87652	19.89448		0.000	4.008984	10.86675
10%—1000 mm/min	35%—12 mm/min			17.23603	0.843997	20.42192	13.22705	21.24502		0.000	4.008984	11.7906
10%—1000 mm/min	35%—120 mm/min			18.5189	0.843997	21.94191	14.50992	22.52788		0.000	4.008984	12.66817
10%—1000 mm/min	35%—1000 mm/min			19.57877	0.843997	23.19769	15.56978	23.58775		0.000	4.008984	13.39319
35%—12 mm/min	35%—120 mm/min			1.282867	0.843997	1.51999	−2.72612	5.29185		0.882	4.008984	0.877567
35%—12 mm/min	35%—1000 mm/min			2.342733	0.843997	2.775762	−1.66625	6.351717		0.414	4.008984	1.602587
35%—120 mm/min	35%—1000 mm/min			1.059867	0.843997	1.255771	−2.94912	5.06885		0.942	4.008984	0.72502

**Table A4:** Power law coefficients for relative elastic modulus.

(i) All effects tested								
SUMMARY OUTPUT								
Regression Statistics								
Multiple R	0.987414891							
R-Square	0.974988167							
Adjusted R-Square	0.949976334							
Standard Error	0.271196411							
Observations	7							
ANOVA								
	df	SS	MS	F	Significance F			
Regression	3	8.600881	2.866960437	38.98108	0.006664711			
Residual	3	0.220642	0.073547494					
Total	6	8.821524						
	Coefficients	Standard Error	t Stat	*p*-value	Lower 95%	Upper 95%	Lower 95.0%	Upper 95.0%
Intercept	−0.875840537	0.948816	−0.92308768	0.424043	−3.895397127	2.143716054	−3.8954	2.143716
ln(VF)*ln(*v**)	−0.175108003	0.098317	−1.78105814	0.172934	−0.487996058	0.137780052	−0.488	0.13778
ln(VF)	0.424507738	0.578737	0.733507366	0.516372	−1.417291192	2.266306667	−1.41729	2.266307
ln(*v**)	−0.064898598	0.157288	−0.41261096	0.707607	−0.565458022	0.435660826	−0.56546	0.435661
(ii) Interaction removed (Model form Equation (8))								
SUMMARY OUTPUT								
Regression Statistics								
Multiple R	0.973930655							
R-Square	0.948540921							
Adjusted R-Square	0.922811381							
Standard Error	0.336878128							
Observations	7							
ANOVA								
	df	SS	MS	F	Significance F			
Regression	2	8.367576	4.183788149	36.86583	0.002648037			
Residual	4	0.453947	0.113486873					
Total	6	8.821524						
	Coefficients	Standard Error	t Stat	*p*-value	Lower 95%	Upper 95%	Lower 95.0%	Upper 95.0%
Intercept	0.5583413	0.623376	0.895673901	0.421056	−1.172426972	2.289109572	−1.17243	2.28911
ln(VF)	1.426756419	0.167927	8.496306304	0.001052	0.96051724	1.892995598	0.960517	1.892996
ln(*v**)	0.182838972	0.091213	2.004536323	0.115517	−0.070407808	0.436085751	−0.07041	0.436086
(iii) *v** removed (Model form Equation (7))								
SUMMARY OUTPUT								
Regression Statistics								
Multiple R	0.947020666							
R-Square	0.896848142							
Adjusted R-Square	0.87621777							
Standard Error	0.426604401							
Observations	7							
ANOVA								
	df	SS	MS	F	Significance F			
Regression	1	7.911567217	7.911567217	43.47222411	0.001206			
Residual	5	0.909956573	0.181991315					
Total	6	8.821523791						
	Coefficients	Standard Error	t Stat	*p*-value	Lower 95%	Upper 95%	Lower 95.0%	Upper 95.0%
Intercept	−0.566488473	0.343823459	−1.647614375	0.160348106	−1.45031	0.317337865	−1.45031481	0.317337865
ln(VF)	1.396374542	0.211785388	6.593346958	0.001205817	0.851963	1.940786212	0.851962871	1.940786212

**Table A5:** Power law coefficients for strain at 0.5 MPa.

(i) All effects tested								
SUMMARY OUTPUT								
Regression Statistics								
Multiple R	0.956551							
R-Square	0.914991							
Adjusted R-Square	0.829981							
Standard Error	0.101006							
Observations	7							
ANOVA								
	df	SS	MS	F	Significance F			
Regression	3	0.3294297	0.109809896	10.76340172	0.040987508			
Residual	3	0.0306065	0.010202155					
Total	6	0.3600362						
	Coefficients	Standard Error	t Stat	*p*-value	Lower 95%	Upper 95%	Lower 95.0%	Upper 95.0%
Intercept	3.701169	0.3533817	10.4735702	0.001858294	2.576550111	4.825786989	2.576550111	4.825786989
ln(VF)	−0.29936	0.2155476	−1.388850876	0.259024529	−0.98533206	0.38660516	−0.98533206	0.38660516
ln(*v**)	−0.01761	0.058581	−0.300684243	0.783289238	−0.20404518	0.168816427	−0.204045178	0.168816427
ln(VF)*ln(*v**)	−0.00232	0.0366176	−0.063339373	0.953480359	−0.1188529	0.114214223	−0.118852895	0.114214223
(ii) Interaction removed (Model form Equation (8))								
SUMMARY OUTPUT								
Regression Statistics								
Multiple R	0.956492							
R-Square	0.914877							
Adjusted R-Square	0.872315							
Standard Error	0.087532							
Observations	7							
ANOVA								
	df	SS	MS	F	Significance F			
Regression	2	0.3293888	0.164694379	21.49538325	0.007245943			
Residual	4	0.0306474	0.007661849					
Total	6	0.3600362						
	Coefficients	Standard Error	t Stat	*p*-value	Lower 95%	Upper 95%	Lower 95.0%	Upper 95.0%
Intercept	3.720165	0.1619734	22.96774395	2.12916E−05	3.270454169	4.169874909	3.270454169	4.169874909
ln(VF)	−0.28609	0.0436329	−6.556721488	0.002798264	−0.40723272	−0.164944259	−0.407232723	−0.164944259
ln(*v**)	−0.01433	0.0237	−0.604769314	0.577962189	−0.08013487	0.051468772	−0.080134869	0.051468772
(iii) *v** removed (Model form Equation (7))								
SUMMARY OUTPUT								
Regression Statistics								
Multiple R	0.952415							
R-Square	0.907094							
Adjusted R-Square	0.888512							
Standard Error	0.081792							
Observations	7							
ANOVA								
	df	SS	MS	F	Significance F			
Regression	1	0.3265865	0.326586468	48.81756903	0.000924648			
Residual	5	0.0334497	0.006689937					
Total	6	0.3600362						
	Coefficients	Standard Error	t Stat	*p*-value	Lower 95%	Upper 95%	Lower 95.0%	Upper 95.0%
Intercept	3.808342	0.0659206	57.77164412	2.93993 × 10^−8^	3.638887485	3.977796111	3.638887485	3.977796111
ln(VF)	−0.28371	0.0406052	−6.986957065	0.000924648	−0.3880858	−0.17932781	−0.388085801	−0.17932781

**Table A6:** Power law coefficients for strain-resolved energy density.

(i) All effects tested								
SUMMARY OUTPUT								
Regression Statistics								
Multiple R	0.997972336							
R-Square	0.995948783							
Adjusted R-Square	0.991897566							
Standard Error	0.15929157							
Observations	7							
ANOVA								
	df	SS	MS	F	Significance F			
Regression	3	18.71364306	6.237881018	245.8394074	0.000437219			
Residual	3	0.076121413	0.025373804					
Total	6	18.78976447						
	Coefficients	Standard Error	t Stat	*p*-value	Lower 95%	Upper 95%	Lower 95.0%	Upper 95.0%
Intercept	12.6162554	0.557302438	22.6380768	0.00018876	10.84267031	14.38984048	10.84267	14.38984
ln(VF)	1.840173177	0.339930385	5.413382441	0.01236314	0.75836298	2.921983375	0.758363	2.921983
ln(*v**)	0.008960023	0.092385415	0.096985258	0.928854237	−0.285051598	0.302971645	−0.28505	0.302972
ln(VF)*ln(*v**)	−0.054965723	0.057747974	−0.95182081	0.411407776	−0.238745551	0.128814104	−0.23875	0.128814
(ii) Interaction removed (Model form Equation (8))								
SUMMARY OUTPUT								
Regression Statistics								
Multiple R	0.997359196							
R-Square	0.994725366							
Adjusted R-Square	0.992088048							
Standard Error	0.157408018							
Observations	7							
ANOVA								
	df	SS	MS	F	Significance F			
Regression	2	18.69065533	9.345327666	377.1732033	2.78218 × 10^−5^			
Residual	4	0.099109137	0.024777284					
Total	6	18.78976447						
	Coefficients	Standard Error	t Stat	*p*-value	Lower 95%	Upper 95%	Lower 95.0%	Upper 95.0%
Intercept	13.06643952	0.291275442	44.85939296	1.47673 × 10^−6^	12.25772924	13.87514979	12.25773	13.87515
ln(VF)	2.154775153	0.07846459	27.4617525	1.0457 × 10^−5^	1.936922526	2.37262778	1.936923	2.372628
ln(*v**)	0.086723886	0.042619552	2.03483807	0.111596729	−0.031606959	0.205054732	−0.03161	0.205055
(iii) *v** removed (Model form Equation (7))								
SUMMARY OUTPUT								
Regression Statistics								
Multiple R	0.994618205							
R-Square	0.989265373							
Adjusted R-Square	0.987118447							
Standard Error	0.200848757							
Observations	7							
ANOVA								
	df	SS	MS	F	Significance F			
Regression	1	18.58806335	18.58806335	460.7823604	4.06929 × 10^−6^			
Residual	5	0.201701117	0.040340223					
Total	6	18.78976447						
	Coefficients	Standard Error	t Stat	*p*-value	Lower 95%	Upper 95%	Lower 95.0%	Upper 95.0%
Intercept	12.53291207	0.161874829	77.42347677	6.81027 × 10^−9^	12.11679958	12.94902457	12.1168	12.94902
ln(VF)	2.14036447	0.099710251	21.46584171	4.06929 × 10^−6^	1.88405111	2.396677831	1.884051	2.396678
